# Built environment and physical activity among adolescents: the moderating effects of neighborhood safety and social support

**DOI:** 10.1186/s12966-019-0898-y

**Published:** 2019-12-18

**Authors:** Venurs H. Y. Loh, Jenny Veitch, Jo Salmon, Ester Cerin, Lukar Thornton, Suzanne Mavoa, Karen Villanueva, Anna Timperio

**Affiliations:** 1 Institute for Physical Activity and Nutrition (IPAN), School of Exercise and Nutrition Sciences, Deakin University, Geelong, Australia; 2 Mary MacKillop Institute for Health Research, Australian Catholic University, Melbourne, Australia; 3 Melbourne School of Population and Global Health, The University of Melbourne, Melbourne, Australia; 4 The Centre for Urban Research, RMIT University, Melbourne, Australia

**Keywords:** Built environment, Walkability, Physical activity, Adolescent, Ecological models, Multilevel analysis

## Abstract

**Background:**

Increasing emphasis has been placed on improving physical activity levels through multilevel interventions. This study aims to examine moderating effects of neighborhood safety (crime and traffic) and social support (from parent and sibling/peer) for physical activity in the relationship between the built environment and moderate-to-vigorous physical activity (MVPA) outside school hours among adolescents in Melbourne.

**Methods:**

Data were from the NEighbourhood Activity in Youth study conducted among adolescents in Melbourne, Australia (*n* = 358, 15.3 (SD = 1.5) years). MVPA outside school hours was assessed by accelerometer. Built environment features within 1 km and 2 km residential buffers including recreation facilities, park area, and walkability and its components were assessed using Geographic Information Systems. Neighborhood safety, social support for physical activity and sociodemographic information were self-reported by adolescents. Multilevel linear regression models were used to estimate associations.

**Results:**

Support for physical activity from sibling/peer positively moderated the relationship between recreation facilities (1 km), residential density (2 km) and MVPA. Recreation facility (count within 2 km), walkability (1 km and 2 km) and residential density (1 km) had significant positive associations with MVPA outside school hours.

**Conclusion:**

The built environment appeared to have stronger facilitating effects on MVPA among adolescents who had favourable support for physical activity from their sibling or peer. Multilevel interventions that target the built environment and social factors are needed to promote MVPA outside school hours among adolescents.

## Introduction

Globally, 80% of adolescents (aged 13–17) do not meet the moderate-to-vigorous physical activity (MVPA) guidelines of 60 min per day [1] and 17% are overweight or obese [2]. Despite numerous interventions developed to promote physical activity, which often focus on individual behavior change, most interventions have moderate-to-small effects on objectively-measured MVPA (average 4 min/day) [3]. Understanding the determinants of physical activity among adolescents, particularly beyond intrapersonal influences, is essential for the development of effective public health interventions to produce long term health benefits.

Socioecological models highlight that there are multiple physical environmental influences on physical activity [4]. The current evidence on the relationship between the built environment and physical activity is less consistent among adolescents than adults [5]. Reviews [5] have reported consistent relationships between some features of the built environment (e.g., availability of recreation facilities and mixed land use) and youth physical activity, but inconsistent associations with other built environment features (e.g., street connectivity and walkability). These inconsistencies may in part be due to the use of different measures of the built environment [6] or due to potential moderation by other factors.

In recent years, there has been an increased emphasis on the role of the social environment as a key modifiable determinant of physical activity [7]. Studies on adults from the United States and older adults from China found significant interactions between some features of the built environment (e.g., walkability and parks) and perceived pedestrian safety on physical activity [8, 9]. For example, activity-friendly built environments were associated with higher physical activity among adults who perceived their neighborhoods to be safer compared to those who perceived their neighborhoods to be less safe [8, 9]. However, in contrast to these findings among adults, only one known adolescent study from the US has examined this relationship and no significant interactions between neighborhood safety and the built environment on physical activity were observed. In addition, social support for physical activity from friends and family was found to be a potential moderator of associations between the built environment and physical activity in adults [10]. However, evidence of interactive effects of walkability and social support on physical activity among adolescents is limited and the findings are mixed: a significant interaction was found among US adolescents [11] but not among Belgium adolescents [12]. The lack of studies and inconsistent findings among adolescents warrants further investigation.

Studies investigating the moderating effects of social factors on the built environment-physical activity relationship are needed to better understand the conditions under which built environment attributes are associated with physical activity, which in turn can help to develop effective multilevel interventions to increase MVPA among adolescents. This cross-sectional study examined whether perceived neighborhood safety (crime and traffic) and social support for physical activity from parents, peers and siblings moderated associations between the built environment and MVPA. It was hypothesized that the positive effect of supportive built environments on MVPA would be stronger among individuals with higher perceived safety and social support.

## Methods

### Study population

This investigation used data collected between August 2014 and December 2015 from the NEighbourhood Activity in Youth (NEArbY) study, which included adolescents living in Melbourne, Australia. It is part of the multi-country IPEN Adolescent (International Physical Activity and the Environment Network Adolescent; http://www.ipenproject.org/IPEN_adolescent.html) project. Ethical clearance was obtained from the Human Ethics Advisory Committee - Health, Deakin University (HEAG-H 152_2013), the Department of Education and Training (2013_002182) and the Catholic Education Office (Project #1950).

### School and participant recruitment

In Australia, a statistical area level 1 (SA1) is the smallest administrative unit used by the Australian Bureau of Statistics (ABS) to release census data, with an average population of ~ 400 individuals [13]. To optimize heterogeneity in built environment and socioeconomic-position, each SA1 across Melbourne was ranked by walkability and income. The walkability index was created in a geographic information system (GIS) based on earlier conceptual work from Frank et al. [14] that combines a standardized sum of street connectivity, land use mix and residential density. Income was based on the median household income within the SA1 from the 2011 census data [15]. Each SA1 was classified into one of four strata: high walkable/high income (HW/HI), high walkable/low income (HW/LI), low walkable/high income (LW/HI) and low walkable/low income (LW/LI), based on median values of walkability and income, respectively.

A total of 137 secondary schools from the four strata were invited to participate in the NEArbY study. Of these, 18 schools agreed to participate (13% school response rate). The schools nominated specific year levels (between years 7 and 12) and interested students received a recruitment package, which included information about the study, a consent form and parent survey. Written parental consent and student assent were received from 528 participants. Of these, 468 students completed an online survey at school and 472 wore an Actigraph accelerometer. Parents also completed a survey (only used in this analysis to provide missing data for age). In total, 465 of those who completed a survey had their residential address successfully geocoded. Based on the SA1 of residential addresses, similar proportions of students in the analytical sample resided in each of the four walkability-income strata (HW/HI = 23%, LW/HI = 25%, HW/LI = 28% and LW/LI = 24%).

### Measures

#### Physical activity outside school hours

MVPA was measured with the ActiGraph GT3X+ accelerometer (a reliable and valid instrument to measure physical activity in youth [16, 17]), worn on the hip for eight consecutive days during waking hours. For some schools, the ActiGraph files were screened at the school on collection using ActiLife and MeterPlus software and some students continued to wear the monitor if wear time was insufficient (i.e., < 4 weekdays with 10 wearing hours; 0 weekend day with 8 wearing hours) to maximize data availability for all purposes. MVPA was defined as the number of minutes spent at ≥4 METS using the Trost et al. [18] age-appropriate cut-points for adolescents. Non-wear periods were determined by ≥60 min of consecutive zeros [19] and subtracted from each 24-h day and period of interest. On weekdays, time spent in MVPA outside of school hours (before school, after school and evenings) was computed for days on which participants had ≥50% wear time [20] in the after school period (end of school to 6 pm). On weekend days, total time spent in MVPA was computed for those with ≥7 h of wear time. Average MVPA and wear time (minutes/day) outside school hours was computed for those with valid data for outside school hours on at least three valid weekdays (outside school hours) and those with at least one valid weekend day. School hours were excluded as it is unlikely that the neighborhood environment would influence MVPA during this time.

#### Objectively measured built environment

The IPEN GIS templates were used to guide the computation of objective indicators of built environments and to ensure comparability across countries [21]. Each participant’s home address was geocoded using ESRI ArcGIS 10.3. Street network buffers of 1 km and 2 km were created around each residential address using street centreline data sourced from VicMap Transport [22] and processed to remove non-walkable roads (freeways, on/off ramps). While there is no consensus on the most appropriate buffer size, 1 km and 2 km represent walkable or threshold distances for adolescents [23], and were prescribed by the IPEN adolescent study GIS guidelines.

#### Recreation facilities

Defined as a count of publicly-funded recreational facilities (e.g., soccer fields, basketball courts) within each buffer. Recreational facility data were compiled from a range of sources [24, 25].

#### Park area

The total park area (m^2^) of all parks that intersected each buffer was calculated. Parks included protected areas, natural and semi-natural areas, parkland and gardens, organized recreation areas, services and utilities areas, civic squares and promenades [24].

#### Walkability and walkability components

For analyses, the walkability index was computed as a sum of three standardized measures from GIS computed at the 1 km and 2 km street network buffers: street intersections, gross dwelling density and land use mix [26]. The total number of street intersections with ≥3 legs within each buffer was calculated using Vicmap Transport [22]. Gross residential density was calculated as the number of dwellings divided by residential area within the buffer (dwelling/m^2^). Meshblock level (smallest geographical area defined by the ABS) residential dwelling data were sourced from the 2011 census [27]. For this analysis, residential density was multiplied by 10,000 so the coefficient is interpreted as one dwelling increase per hectare. Within each buffer, the area of four land uses (residential, commercial, entertainment and institutional) were extracted to compute land use mix. Land use data were compiled from a range of sources (Axiom Business Points [28]; 2010 Victorian Valuer General’s Office valuations database [29]; Metro ARIA TAFE locations [30]; National Health Service Directory [31]; and VEAC Public Open Space Inventory [24]). The formula for land use mix was provided by Giles-Corti et al. [26]. The land use mix score ranges between 0 and 1. A score of 0 indicates that a buffer has a single land use and a score of 1 indicates that the area has an even distribution of all land uses.

### Potential moderators

#### Perceived safety from crime

Perceived safety from crime was measured using modified scales from the Neighborhood Environment Walkability Scale-Youth (NEWS-Y) questionnaire, which has acceptable reliability (test-retest intraclass correlation coefficients [ICC] = 0.73 to 0.75) [32]. Participants were asked to respond to seven statements on a 4-point Likert scale (1 = strongly disagree; 4 = strongly agree) about the level of crime in their neighborhood and fear of abduction or attack by strangers around home, outside with friends on local streets, and in a local/nearby park. Summary scores were computed by averaging the scores on the corresponding items (reverse coded where necessary in the direction consistent with higher safety from crime). Internal consistency evaluated with the current sample was α = 0.85, which was similar to that observed in Hong Kong adolescents (α = 0.82) [32].

#### Perceived traffic-related safety and pollution

The perceived traffic-related safety and pollution measure was also adapted from the NEWS-Y questionnaire, which has acceptable reliability (ICC = 0.67 to 0.81) [32]. Participants were asked to respond to eight statements on a 4-point Likert scale (1 = strongly disagree; 4 = strongly agree) about the level of safety from traffic in their neighborhood. These items covered the amount and speed of traffic on nearby streets, exhaust fumes, street lighting, visibility of walkers and cyclists from home and pedestrian crossings and traffic lights. An additional item relevant to adolescents’ concern on traffic safety was included in the NEArbY survey asking if participants feel safe crossing the streets in their neighborhoods. Summary scores were computed by averaging the scores on the corresponding items (reverse coded where necessary).

#### Perceived parent support for physical activity

Parent support was assessed by four items pertaining to encouragement of physical activity, provision of transportation, co-participation in physical activity and payment for sporting clubs (adapted from Norman et al. [33] for the IPEN Adolescent Study [32], with acceptable reliability (ICC = 0.79) [32]). Frequency of parent support was rated on a 4-point Likert scale ranging from never (coded 0) to very often (coded 4). Scores for each item were summed. Total scores could range from zero to 16. Internal consistency of the items evaluated with the current sample was higher (α = 0.81) than observed in a previous study (α = 0.68) [32].

#### Perceived sibling or peer support for physical activity

The siblings or peer support scale was also adapted from Norman et al. [33] and included two items that assessed (a) companionship for physical activity and (b) offers to walk or ride to school or friend’s house. Frequency of social support was rated on a 4-point Likert scale ranging from never (coded 0) to very often (coded 4). Scores for each item were summed. Total scores could range from zero to eight. The test-retest reliability of this scale was acceptable (ICC = 0.74) [32]. Internal consistency of the items evaluated with the current sample was higher (α = 0.72) than in a previous study (α = 0.69) [32].

### Covariates

Age (years) and sex were self-reported by the adolescents. Missing information on adolescent age was supplemented from the parents survey (*n* = 7). Information on residential neighborhood disadvantage was obtained from the ABS SEIFA Index of Relative Socioeconomic Disadvantage [34], reflecting the overall level of disadvantage at the SA1 level.

### Statistical analyses

Of the 465 participants, those who did not provide valid accelerometer data (*n* = 97), or information about age (*n* = 5) and sex (n = 5) were excluded. This reduced the analytic sample to 358 participants.

First, separate multilevel linear models were conducted to examine associations between each built environment variable and MVPA, accounting for age, sex, neighborhood disadvantage and accelerometer wear time. School ID and neighborhood SA1s were entered as random effect variables to account for cross-classified clustering. Moderating effects of neighborhood safety (crime and traffic) and social support (parent, sibling/peer) on associations between the built environment and MVPA were then estimated by adding a two-way interaction term to the main effects in the first step for each built environment exposure separately. Third, significant moderation effects were probed by estimating associations at meaningful values of moderators (mean ± 1SD) and were presented graphically (predicted MVPA [minutes/day] plotted against the minimum and maximum of built environment variables at meaningful values of the moderators). Data analyses were undertaken using STATA/SE 15.0.

## Results

The mean age was 15.3 (SD = 1.5) years and 59% were girls. On average, participants spent 25.1 min/day (SD = 14.9) in MVPA outside school hours on weekdays and 24.3 min/day (SD = 20.7) in MVPA on weekend days. There were no significant differences in MVPA accumulated outside school hours on weekdays and MVPA accumulated on weekend days (*p* = 0.56). The average wear time outside school hours on weekdays and weekend days were 441 and 1317 min per day, respectively.

Table 1 presents descriptive statistics for each objectively-assessed built environment variable and scores for self-reported safety from crime, traffic-related safety and pollution, parent support and sibling/peer support. Walkability and land use mix had similar mean values within the 1 km and 2 km buffer, residential density had lower mean values in the 2 km buffer than the 1 km buffer, while the remaining environmental variables had higher mean values in the 2 km buffer than the 1 km buffer.

**Table 1:** Descriptive information for the analytical sample

		1 km buffer			2 km buffer	
	N	Mean (SD)	Range		Mean (SD)	Range
*Objectively assessed built environment variables*						
Recreation facilities (n)	358	1.4 (1.7)	0.0–11.0	5.2 (5.0)	0.0–30.0	
Park area (km^2^)	358	0.4 (0.8)	0.0–5.8	1.2 (1.3)	0.0–9.7	
Walkability score^a^	358	−0.1 (2.2)	−5.7- 10.0	−0.1 (2.3)	−6.7-9.6	
Street intersection (n)^b^	358	88.8 (41.9)	6.0–289	349.1 (163.2)	25.0–1209.0	
Residential density (dwelling/ha)	358	25.7 (8.3)	3.9–60.4	20.6 (6.8)	1.8–42.0	
Land use mix^c^	358	0.4 (0.2)	0.0–0.9	0.5 (0.1)	0.0–0.9	
		Mean (SD)	Range			
*Potential moderators*						
Perceived safety from crime^d^	358	3.4 (0.6)	1.3–4.0			
Perceived traffic-related safety and pollution^e^	357	3.0 (0.4)	1.5–4.0			
Perceived parent support for PA^f^	357	9.2 (4.1)	0.0–16.0			
Perceived sibling/peer support for PA^g^	355	3.7 (2.2)	0.0–8.0			

Note: *SD* Standard deviation, *PA* Physical activity; ^a^standardized sum of street intersections, residential density and land use mix; ^b^count of three or more way street intersections; ^c^mix of land use based from 0 to 1; ^d^higher value indicates safest from crime; ^e^higher value indicates safest from traffic-related safety and pollution; ^f^higher value indicates better parent support for physical activity; ^g^higher value indicates better sibling/peer support for physical activity

**Fig. 1:**
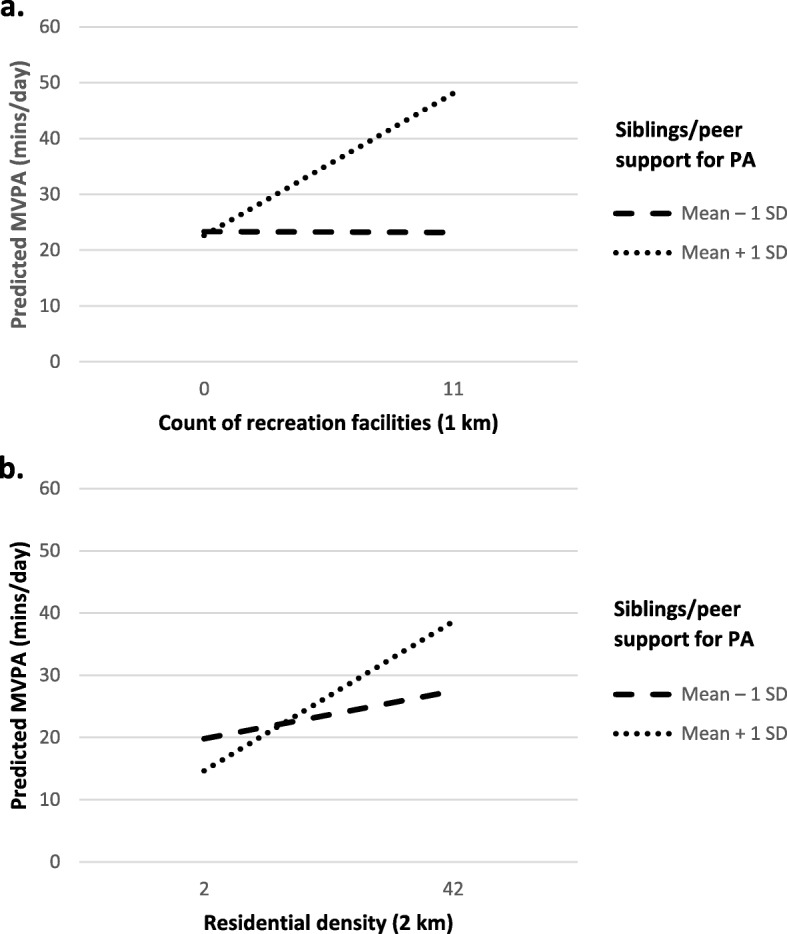
Marginal means plot of significant interactions between a. recreation facilities within 1 km (*n* = 356) and b. residential density within 2 km (*n* = 356) and moderate-to-vigorous physical activity (MVPA) according to sibling/peer support among boys with a mean age of 15

Sibling/peer support was the only moderator of associations between the objective built environment variables and MVPA outside school hours (Table 2). In general, there were stronger positive associations between recreation facilities within 1 km and residential density within 2 km and MVPA for those with higher levels of sibling/peer support, compared to those with less support (Fig. 1). For recreation facilities within 1 km, associations were b = − 0.01 (95% CI: − 1.07, 1.06, *p* = 0.9) for participants with lower and b = 2.19 (95% CI: 0.86, 3.52, *p* = 0.001) for participants with higher sibling/peer support. For residential density within 2 km and MVPA, associations were b = 0.19 (95% CI: − 0.10, 0.50, *p* = 0.50) at lower and b = 0.58 (95% CI: 0.26, 0.91, p = < 0.001) at higher sibling/peer support. Parent support for physical activity, perceived crime and traffic-related safety and pollution did not moderate associations between the built environment and MVPA.

**Table 2:** Associations between built environment (1 km and 2 km network buffers) and moderate-to-vigorous physical activity (MVPA) outside school hours (minutes/day) among adolescents (B coefficient and 95% confidence intervals)

*N* = 358		MVPA outside school hours (mins/day)		
	Adjusted B (95%CI)^a^	Moderator^b^	Adjusted B (95%CI)^a^	Moderator^b^
Built environment	1 km buffer		2 km buffer	
Recreation facilities	0.74 (−0.13, 1.63)	Sibling/peer support	0.55 (0.23, 0.87)***	–
Park area	0.27 (−1.51, 2.05)	–	1.11 (−0.01, 2.33)	–
Walkability	0.77 (0.02, 1.51)*	–	0.79 (0.06, 1.51)*	–
Street intersection	0.01 (−0.02, 0.05)	–	0.01 (−0.01, 0.01)	–
Residential density	0.20 (0.01, 0.39)*	–	0.37 (0.12, 0.62)**	Sibling/peer support
Land use mix	5.61 (−1.67, 12.9)	–	4.12 (−5.21, 13.47)	–

Notes: *CI* Confidence interval; ^a^ Adjusted for age, sex, neighborhood disadvantage and accelerometer wear time; ^b^ Each interaction term added to model – significant (*p* < 0.05) interaction terms noted. * *p* < 0.05; ** *p* < 0.01; *** *p* < 0.001

Main effect associations for remaining variables are shown in Table 2. Within 1 km, walkability and residential density were positively associated with daily MVPA outside of school hours. Within 2 km, recreation facilities and walkability were positively associated with daily MVPA outside of school hours. There were no significant associations between MVPA and park area, street intersections or land use mix for either buffer size.

## Discussion

The main aim of the study examined whether the associations between built environment features and objectively-assessed MVPA were moderated by neighborhood safety and social support for physical activity. Overall, two aspects of the built environment appeared to have stronger facilitating effects on MVPA among adolescents who had favorable support from their siblings or peers. Walkability within 1 and 2 km, residential density within 1 km and recreation facilities within 2 km showed positive associations with MVPA that were not moderated by neighborhood safety or social support.

Our findings showed that built environment features had interactive effects with social support on MVPA. Positive associations between recreation facilities, residential density and MVPA were found among those with the highest sibling/peer support. This pattern of interactions suggests that while the built environment can support MVPA, the relationship may vary according to the perceived level of social support. This is not surprising given that adolescents are not totally independent and may rely on others for encouragement for MVPA opportunities, particularly outside school hours. Our finding is in agreement with a previous study among US adolescents which found a positive interaction between walkability and social support on MVPA [11].

Contrary to our hypothesis, adolescents’ perception of safety from crime and traffic did not moderate associations between the built environment and MVPA. The small variation and high mean scores for neighborhood safety suggests potential ceiling effects. Further, although the sample was drawn from areas of different socioeconomic status, the small variation in neighborhood safety scores may have limited the ability to observe statistically significant interactions, leading to an underestimation of interactions between individual exposure and the built environment.

Our findings showed positive, albeit relatively weak associations between recreation facilities (2 km), walkability (1 and 2 km), residential density (1 km) and MVPA outside school hours. The association between recreation facilities and MVPA is consistent with previous studies conducted among adolescents [35, 36]. The positive association between residential density, walkability and MVPA among adolescents is also consistent with studies from New Zealand [37] and the U.S. [38]. However, no significant associations were observed between park area, street connectivity, land use mix and MVPA outside school hours. The non-significant relationship between street connectivity and MVPA is consistent with two previous studies among adolescents [39, 40] but other studies have reported mixed findings [37, 41]. Greater street connectivity tends to indicate a shorter travel route between origins and destinations, and may therefore be more relevant to transport-related physical activity than overall MVPA [42]. The null association between land use mix and MVPA could be due to limitations in measurement. Although widely used, the land use mix measure lacks specificity, which makes it difficult to identify actual destinations, as well as the quality of the built environment within the neighborhood. For example, two neighborhoods could have the same land use mix score but have very different destinations that may impact physical activity differently. Our study also found no association between park areas and MVPA, though potentially different findings could have been found if an alternative method of computing park area was used (e.g., park area within the buffer only). However, a previous observational study on park visitations in Australia [43] found few adolescents were observed in parks, and those present were mainly observed engaging in sedentary or low intensity activities, such as sitting or standing. Therefore, stronger associations may be observed between park area and light-intensity activity.

Our study explored associations of the built environment with MVPA using two buffer sizes. Many studies on the built environment and physical activity have used a 1.6 km (1 mile) buffer size, as this is a comfortable walking distance for adolescents [44]. However, researchers have cautioned the use against a single buffer size as the concept of reasonable walking distance may vary by age group [6, 44]. Given our sample was adolescents, and the count of certain built environment variables within 1 km buffers was small, the 2 km buffer seemed to be a more sensitive buffer size to understand the effect of the built environment and MVPA in a city such as Melbourne. Defining and choosing the most appropriate buffer size to measure meaningful differences in the built environment and health behavior is an ongoing challenge.

### Limitations

The cross-sectional nature of the study means that claims about causality are not possible. Longitudinal studies or natural experiments would add strength to the study findings. The accelerometer-assessed MVPA measure lacked specificity as it was not possible to distinguish between activity undertaken in the neighborhood or elsewhere and the MVPA assessed may not have occurred within the neighborhood. The MVPA measure was also unable to discriminate between physical activity domains (e.g., leisure or transport), which may lead to the conceptual mismatch between the built environment and physical activity. It is therefore likely that the associations we reported in this study are underestimated. Incorporating data from Global Positioning Systems as well as domain-specific information would help identify where and which domain of physical activity have occurred, which are important considerations for future research. In addition, it is possible that physical activity accumulated in the neighborhood may not meet the intensity needed to be defined as MVPA using the criteria applied in this study, which may explain the weak associations found. The lack of conceptual match between physical activity domain and environmental attributes information is subject to bias associated with conceptual mismatch. Further, the GIS-computed built environment measures were unable to capture the quality, conditions and actual destinations (particularly for land use mix), which have been shown to be important for physical activity among adults and older adults [45]. The exact information on response rate was unavailable for the study and this may have implications on sample generalizability. Finally, data on perceived neighborhood safety and social support variables were self-reported by adolescents, and therefore subject to recall and/or desirability bias [46].

## Conclusion

Some features of the neighborhood built environment have the potential to support MVPA among adolescents. Of all life stages, adolescence is the period with most profound changes, including changes in the focus of social relationships from parents to peers. Strongest associations between the built environment and MVPA were found among those with the highest level of social support from sibling or peer. These findings imply that multilevel interventions that target both the built environment and social support may be needed to maximize active behaviors among adolescents outside school hours.

## Data Availability

The dataset used and/or analyzed during the current study are available from the corresponding author on reasonable request.

## Abbreviations

ABS: Australian bureau of statistics
GIS: Geographic information systems
ICC: Intraclass correlation coefficients
IPEN: International physical activity and the environment network adolescent
MVPA: Moderate-to-vigorous physical activity
NEArbY: NEighbourhood activity in youth
PA: Physical activity
SA1: Statistical area level 1
SD: Standard deviation
